# Condom failure and pre-exposure prophylaxis use experience among female sex workers in Ethiopia: a qualitative study

**DOI:** 10.1186/s12889-022-13468-3

**Published:** 2022-05-31

**Authors:** Minilik Demissie Amogne, Eduard J. Sanders, Wudinesh Belete Belihu, Jesper Sundewall, Anette Agardh

**Affiliations:** 1 Department of Clinical Sciences, Social Medicine and Global Health, Lund University, Malmö, Sweden; 2 Ethiopian Public Health Institute, Addis Ababa, Ethiopia; 3 KEMRI/Wellcome Trust Research Programme Centre for Geographic Medicine Research–Coast, Kilifi, Kenya; 4 Nuffield Department of Medicine, University of Oxford, Headington, UK; 5 Department of Global Health, University of Amsterdam, Amsterdam, The Netherlands; 6 Kenya Medical Research Institute, Kilifi, Kenya; 7 HEARD, University of KwaZulu-Natal, Durban, South Africa

**Keywords:** Condom use, PrEP challenge, Side effect, FSWs, Ethiopia

## Abstract

**Background:**

Female sex workers (FSW) remain a highly exposed group for HIV/STIs due to different factors including condom failure. In Ethiopia, pre-exposure prophylaxis (PrEP) has recently been introduced as an intervention strategy to prevent new HIV infections, but knowledge about FSWs’ experiences of condom failure and PrEP use remains scarce. Therefore, this study explores FSWs’ experiences concerning condom failure and their attitudes towards, and experiences of, PrEP uptake.

**Method:**

A qualitative study using in-depth interviews was conducted among FSWs in Addis Ababa. A manifest and latent content analysis method was applied to identify categories and emerging themes.

**Result:**

Seventeen FSWs (10 who started on PrEP, 1 who discontinued, and 6 who didn’t start) were interviewed. FSWs described the reasons behind condom failure, the mechanisms they used to minimize the harm, and their attitudes towards PrEP use. FSWs struggled with the continuous risk of condom failure due to factors related to clients’ and their own behavior. PrEP was mentioned as one the strategies FSWs used to minimize the harm resulting from condom failure, but PrEP use was compounded with doubts that deterred FSWs from uptake. FSWs’ misconceptions, their lack of confidence, and PrEP side effects were also mentioned as the main challenges to start taking PrEP and/or to maintain good adherence.

**Conclusion:**

The demands and behavior of the clients and FSWs’ own actions and poor awareness were factors that increased the exposure of FSWs to condom failure. In addition, the challenges associated with PrEP uptake suggest the need for user-friendly strategies to counteract these barriers and facilitate PrEP uptake.

**Supplementary Information:**

The online version contains supplementary material available at 10.1186/s12889-022-13468-3.

## Introduction

Female sex workers (FSW) are highly vulnerable to sexually transmitted infections (STI), HIV, and unintended pregnancy due to a number of adverse circumstances, including large numbers of sex partners, unsafe working conditions, inconsistent condom use, frequent alcohol and other substance use, exposure to violence, and economic deprivation [1–4]. According to a meta-analysis from 111 studies worldwide, FSWs are 13.5 times more likely to be infected by HIV than other women of reproductive age [5]. Moreover, FSWs often have little ability to mitigate these challenges because of social marginalization and criminalized work environments [3].

Condom failure is when breaking, leaking, or slipping-off occurs during penetrative sexual activity, and such failure is a frequent experience among FSWs [6–8]. In a study among 4,886 FSW in Ethiopia, 25.5% reported condom breakage/slippage at least once in the past month preceding the survey [9]. In the aforementioned study, heavy episodic drinking among FSWs was found to be one of the factors that increased condom breakage/slippage [9]. Further, a study among 291 FSWs conducted in India also found that lack of experience and incorrect handling of the male condom contributed to condom breakage [10]. Studies among FSWs in China (*N* = 200) and South Africa (*N* = 100) also found that poor condom fit, the duration and roughness of the sex act, and the use of drugs and alcohol contributed to condom breakage/slippage [11, 12]. To minimize the incidence of condom failure, interventions including learning about proper condom use and applying condoms oneself, have been recommended [13].

Daily oral pre-exposure prophylaxis (PrEP) is recommended for all FSWs and HIV-negative partners of serodiscordant couples in Ethiopia since 2020 [14]. Ethiopia has an estimated 2,884 PrEP users, a low rate of 0.3 per 10,000 population compared to 15.5 per 10,000 population in Kenya [15]. Currently, the Ministry of Health has launched a national scale-up of the service to be offered at all ART-providing health facilities and drop-in center clinics. PrEP is a relatively new intervention offered to FSWs and could potentially reduce their risk of HIV infection due to condom failure. However, knowledge is currently scarce concerning FSWs’ attitudes towards and experiences of PrEP use in Ethiopia. Although findings are available from studies conducted in different sub-Saharan countries [16–18] on the acceptability and barriers of PrEP uptake that might serve as a resource for Ethiopia, it is important to generate country-specific knowledge on how to promote and sustain PrEP uptake.

Thus, in order to better support FSWs’ HIV prevention efforts in a setting where PrEP is becoming available, increased knowledge is needed about FSWs’ experiences of condom failure and how they view PrEP use in this regard. To our knowledge, this is the first study exploring the experiences of FSWs with regard to condom failure and daily oral PrEP in Ethiopia. The aim of the study was to explore FSWs’ experiences concerning condom failure and their attitudes towards and experiences of PrEP use.

## Material and methods

### Study setting and design

The study was conducted in Addis Ababa, the capital of Ethiopia, with an estimated population of over 4 million [19]. Sex work takes place on the streets and in bars, hotels, red light houses, massage houses, and small establishments that sell local drinks.

The study participants were invited to the study using the snowball sampling method where the first interviewee was contacted through the drop-in center irrespective of their PrEP taking status. The study was conducted from January to March 2021. FSWs are a hard-to-reach population; therefore, we used a Population Service International (PSI) drop-in center since this NGO had established a good relationship with this group. At the time of data collection PSI was the only organization with drop-in center in Addis Ababa. Drop-in centers (DICs) are community-based centers, aiming at offering confidential, comprehensive, and anonymous services for female sex workers including: HIV testing, STI diagnosis and treatment, family planning and risk reduction counselling [20]. Inclusion criteria for participation in the current study were aged 18 years or older, currently engaged in sex work, and written informed consent prior to the study. Each participant was invited to a private location for an interview that lasted an average of 45 min. Permission was obtained from each study participant to audio record the interview. The data collection process was finalized after reaching a consensus among the research team members on saturation of the information. After reading through the transcribed interviews, the team members noted that the information flow was becoming repetitive, with no additional new points forthcoming, which led to the decision to finalize the data collection.

The interviews were conducted by the first and third author, who were experienced in sexual and reproductive health matters and fluent in the local language (Amharic). They used a semi-structured interview guide (Supplementary document 1) that included participants’ demographic characteristics and views about their working situation, condom use, alcohol consumption, HIV and sexually transmitted infection risks, and PrEP. Four pilot interviews were conducted to test the flow of the interview and the in-depth interview guide. The pilot interviews were used to modify and revise the interview guide. The final two pilot interviews utilized the revised interview guide and were therefore included in the analysis. After completion of the interview, each participant was compensated with ~ US$8 (300 Eth-birr) for transportation and time spent during the interview.

### Data analysis

Data analysis was performed according to manifest and latent qualitative content analysis as described by Graneheim and Lundman [21]. The unit of analysis was the interview, and the interview texts were sufficiently rich to allow for an exploration of the latent level. First, the recorded interview was transcribed in Amharic and then translated into English. Both interviewers read through each transcription independently and identified sentences that had a particular meaning (i.e. meaning units). The meaningful units were then coded manually by the first author. Codes were grouped into categories and subcategories based on similar ideas. Field notes were taken during the interviews and used to clarify some of the context related texts.

The two interviewers discussed the resulting categories until they reached a consensus. Then the categories were shared with the other co-authors for further discussion. Finally, a thorough discussion of the categories among all the authors facilitated the identification of emergent themes.

### Ethical considerations

The study protocol was approved by the Ethiopian Public Health Institute review board (EPHI-IRB). FSWs are one of the vulnerable groups highly exposed to stigma and discrimination. Thus, extra care was taken to maintain the confidentiality and privacy of the study procedure; all personal identifiers were removed and then replaced with code numbers. In addition, individual informed consent was sought from each participant before the interview.

## Results

A total of 17 FSWs participated in the study, with a median age of 25 years (range: 21–35). All but one participant had primary school or no formal education. Ten participants reported taking PrEP, one participant had stopped taking PrEP, and six participants never started it (Table 1). Three themes emerged during the analysis: 1) Struggling with the continuous risk of condom failure, 2) Doubting the feasibility of PrEP as a protection strategy, and 3) Being challenged by the negative aspects associated with PrEP use. The first theme was supported by three categories: a) Difficulties associated with clients’ behavior, b) Dealing with their own inadequacies, and c) Minimizing harm due to condom failure. The second theme was supported by two categories: a) Misconceptions and barriers towards taking PrEP, and b) Lacking confidence in self-efficacy. The third theme was supported by three categories: a) Having to deal with adverse side effects, b) Facing risk of stigma, and c) Feeling challenged by adherence needs (Table 2). The details of each theme and category are discussed below where the themes are shown in bold, and the categories are underlined.

**Table 1:** Characteristics of FSW study participants, Addis Ababa, Ethiopia, 2021

Characteristic	Frequency
**Median age = 25 years (range: 21–35)**	
**PrEP uptake status**	
Number of FSWs on PrEP	**10**
Discontinued taking PrEP	**1**
Not started PrEP^a^	**6**
**Educational status**	
No formal education	**5**
Some grade of elementary school	**11**
Secondary and above	**1**
**Marital status**	
Never married	**12**
Divorced	**5**
**Number of children**	
0	**8**
1	**7**
2	**2**
**Current meeting location of clients**	
Street	**14**
Bar and/or street	**3**

^a^One FSW was HIV (self-identified) positive so was not offered PrEP

**Table 2:** Overview of the themes, categories and sub-categories concerning FSWs’ experience of condom failure and PrEP use

Theme	Categories	Sub-Categories
Struggling with the continuous risk of condom failure	Difficulties associated with clients’ behavior/demand	Deliberate condom breakage/ slippage and piercing
		Men’s focusing only on sexual satisfaction leading to struggle (rough sex) and taking a long time
		Drunk clients
		Poor quality of condom brought by clients
		Size of penis
	Dealing with their own inadequacies	FSWs getting drunk
		Getting involved (FSWs) in the sex (level of absorption)
		Letting a client have sex in different positions/ allowing clients to take control over the position
		Not having proper condom use knowledge/skill
		Negligent FSWs face frequent breakage/slippage
	Minimizing harm due to condom failure	Changing condoms
		Getting rid of harmful fluids
		Taking PrEP
Doubting the feasibility of PrEP as a protection strategy	Misconceptions and barriers towards taking PrEP	Perceiving PrEP as an alternative, and not supplement, to condom use
		Perceiving no difference between taking PrEP daily and taking ART after being positive
		Not liking to take PrEP everyday
		Doubting effectiveness of PrEP for HIV protection
	Lacking confidence in self-efficacy	Fear of being negligent regarding condom use with client
		Fear of forgetting to take the pill while drunk as a reason not to start taking PrEP
		Fear of not being able to manage the side effects
Being challenged by the negative aspects associated with PrEP use	Having to deal with adverse side effects	Having nausea and nightmares; gastritis and back pain; bloating, and sometime diarrhea
	Facing risk of stigma	Being seen as HIV positive when taking PrEP
		The PrEP container is similar to ART
	Feeling challenged by adherence needs	Worrying that drinking and using khat might interfere with PrEP adherence
		Forget to take PrEP in the morning due to sleeping

### Struggling with the continuous risk of condom failure

Female sex workers encountered several risks during their work and these risks mainly emanated from the demand and actions of the clients or from the action and/or reluctance of FSWs. One of the main risks in the everyday life of a FSW was condom use failure, which was primarily due to condom breakage/slippage.

#### Difficulties associated with clients’ behavior/demand

Condom failure was partly due to factors associated with the client’s behavior. Some of the clients who demanded sex without a condom even tried to break/slip/tear the condom deliberately. It was mentioned that when sexual intercourse takes a long time, some clients might attempt to break the condom deliberately to finish sooner.

“There are guys who do not want to use condoms. I experienced deliberate condom breakage; the guy tears it with his finger while we are having sex and tries to continue, but I shout and make him stop. [P 08, age 25]

Another FSW mentioned that those clients who know their status (HIV) might break the condom deliberately because they do not care. When asked “why do you think he starts to tear the condom?” she replied: “*I don’t know, maybe to finish the sex or he already knows his status (HIV) and might not care.” [P 03, age 23].*

To cope with deliberate tearing, FSWs tried to maintain control by putting on the condom for clients. By doing so, they could be able to manage proper condom use and avoid deliberate tearing. One FSW said that *“I put the condom on clients because some men try to tear it”.* The other strategy reported was to identify clients who try to break condoms deliberately and avoid going with them when they come for the second time.

The other reason mentioned which compromised condom use was the client’s focus solely on sexual desire leading to rough sex and condom breakage. One of the participants described this as follows:

“You know, it happens when you struggle. Some men do not think about their health; they only follow their feeling, and when they struggle the condom bursts, although I put it on him properly. When it happens, I often go to a clinic for testing and until now I am healthy, thanks to God”. [P 16, age 25]

On the other hand, some drunk clients took too long a time during sex, causing the condom to break or slip.

“Yes, I have faced a drunken client and most of the time they nag you and give you a hard time. Their behavior changes from time to time and when they have sex, they don’t do it properly. Therefore, I always try to control everything. During sex, they might last long which might cause condom breakage.” [P 17, age 23]

In addition, the quality and storage of the condom were also mentioned as reasons for condom breakage/slippage. In particular, the condom that the client brings was believed to have low quality due to poor storage and also subjected to deliberate tearing. Furthermore*,* condom use could be compromised by the size of the penis. FSWs experienced that the client’s penis size could cause the breakage, or due to the small size of the penis, the condom slips.

“Sometimes when a client takes a too long time it breaks and slips if the client’s penis size is small. I had a client with a small size and I try several times, but the condom does not fit, so I quit going with him.” [P 03, age 23.

#### Dealing with their own inadequacies

FSWs’ own behavior and inadequacies also contributed to condom use failure. One of the issues mentioned was alcohol consumption among the FSWs. According to the FSWs, most of the time it was possible to control the condom use with a drunk client if the FSW was not drunk, but if she was drunk too, it was impossible to use a condom let alone to manage proper condom use. As described by one of the participants:

“I don’t remember the whole thing; in the morning there was a condom on the ground (she laughs). Most of the time if I get drunk, I do not go with a client; I do not trust myself”. [P 02, age 30]

The active involvement of FSWs in the sexual intercourse was also mentioned as a contributor to condom breakage/slippage and also could make FSWs unable to respond to the breakage in time. Thus, when FSWs were actively engaged in the sexual intercourse, they might be less conscious of the condom. In addition, allowing clients to take control over the position during sex was also mentioned as a reason for compromised condom use. Trying to maintain awareness during sexual intercourse with a client and taking action when the condom breaks/slips were mentioned as strategies to control condom use.

“If you let them have different positions of sex and give them freedom, you might also get involved emotionally in the sex and compromise the condom use. So I do not allow that; I only have normal sex position”. [P08, age 25]

The other reason mentioned for breakage and slippage was lack of proper condom use knowledge by FSWs. Most of the time FSWs put the condom on for the client to assure proper condom use, but sometimes due to poor knowledge they faced frequent condom breakage. They mentioned that especially when starting to sell sex, most of them might not know how to use condoms properly, leading to condom use failure. In addition, it was mentioned that FSWs who were negligent could face frequent condom breakage/slippage. Thus, providing education on proper condom use especially for new FSWs was mentioned as one of the main prevention activities. In particular, continuing the support provided by the DIC system was mentioned as the preferred way of reaching FSWs.

“The support should continue with the DIC system. Female sex workers have to get awareness training; they come from different lifestyles and also go through different things so they need to get an education. Currently, the DICs are not operating as before, so they should continue educating FSWs like they did previously. Not only for the current FSWs but also for the future generation. I am watching the new generation of young girls joining the sex work; they only see the money they get; they have no idea what’s coming next. Therefore, they need to get an education, it should continue.” [P 06, age 25]

#### Minimizing harm due to condom failure

Despite FSWs’ actions to avoid condom failure, they sought to minimize the harm done after condom breakage/slippage occurrence. The first thing they did was to change the condom swiftly when it broke during sexual intercourse. The other action to minimize the harm after condom breakage was to either wash, jump and/or urinate to get the fluid out of the body.

“When breakage happens, I try to urinate right away to wash out the fluids” [P8, age 25,]

Furthermore, taking PrEP was also reported by some FSWs to minimize the chance of contracting HIV.

“While I am at work, I might face condom breakage and contract HIV and that worries me. I want to build another life out of sex work, being in this life worries me. So, I try to use condoms properly and also use drugs (PrEP)”. [P 12, age 25]

### Doubting the effectiveness of PrEP as a protection strategy

Different factors contributed to FSWs’ feelings of doubt about PrEP, leading some to avoid taking PrEP. Doubts about PrEP as a protection strategy were fueled by misconceptions and barriers towards taking PrEP and by lack of confidence.

#### Misconceptions and barriers towards taking PrEP

Some FSWs did not understand the role of PrEP and condom use; they considered PrEP as an alternative to condom use, so they chose condoms over PrEP.

“When I ask the nurse the advantage of the PrEP during condom breakage, she said that it is as good as the condom and my understanding was, if it is only as good as the condom, then the condom is enough. So I didn’t start it.” [P 08, age 25]

Another misunderstanding was comparing taking PrEP with taking ART for HIV. The fact that PrEP was taken daily was compared with ART for HIV, which is also being taken on a daily basis. However, some FSWs did not understand that PrEP is just for risk periods only.

“Some women say, if we are going to take it daily, what is the difference between being HIV positive and taking ART? So they say we will take the treatment when we become positive.” [P 12, age 25]

The burden to take a daily pill was mentioned by some as a barrier not to start, while the effectiveness of PrEP for HIV protection was also doubted.

“I don’t know, I do not want to start it, taking it every day……Can it really protect from HIV?” [P15, age 22]

Nevertheless, not all FSWs misunderstood the advantage of PrEP; one FSW when asked about being reluctant to use condoms due to taking PrEP replied as follows:

*Never, it is the reverse for me, the drug gives me strength to be confident about using condoms. I think about my child, then I think about myself, if something happened to me [P 05, 25].*

#### Lacking confidence in self-efficacy

Some FSWs mentioned reasons not to start taking PrEP that emanated from poor confidence and/or self-doubt. FSWs reported that PrEP could make them more reluctant to use condoms and might make them accept being offered sex without a condom. In addition, the possibility of forgetting PrEP, especially during alcohol consumption, was also mentioned as a reason not to start taking it.

“I don’t know, I am afraid of taking it. It might also make me negligent about condom use. If I use the PrEP, when the client requests me, I might go without a condom. In addition, I might forget to take it when I get drunk so to avoid that, I prefer not to start taking the drug.” [P 02, age 30]

Some FSWs believed that the side effects of PrEP could be greater than the protection they obtained from it. In addition, fear of not being able to manage the side effects was mentioned as a reason; although they had not started taking PrEP, what they heard from other FSWs about the side effects of taking PrEP made them fear starting it.

“Because I was afraid of the drug’s side effect. I asked a woman who started to take it and she said it gives nausea, vomiting and also gastric pain. So I decided not to take the drug.” [P 08, age 25]

### Being challenged by the negative aspects associated with PrEP use

FSWs with experience of PrEP described a number of negative aspects which they perceived as challenging, and for some, these were reasons to stop taking it.

#### Having to deal with adverse side effects

Among those FSWs who had taken or were currently taking PrEP, adverse side effects were experienced as a challenge. The reported side effects occurred during the starting period of taking PrEP (approximately 15 days). Having nausea, gastritis, bloating, nightmares, back pain, and sometimes diarrhea were the side effects reported. One FSW had stopped taking it due to the pain in the stomach and other FSWs mentioned that they were going to quit or had thought about quitting.

“It is for the prevention of HIV; it has been 3 months since I started taking it. When I start taking, I used to feel nausea, vomiting and also bloating and false alarm to go to the bathroom, and sometimes diarrhea too. Then I go to the clinic and told them that I was going to quit but they told me that it is temporary. They also told me not to quit unless I get out of this life. As they say, the pain was only for 15 days, then it stops.” [P 03, age 23]

#### Facing risk of stigma

The attitude and perception of other FSWs towards PrEP appeared to be one of the challenges. The similarity of the PrEP and ART containers opened the door for pre-judgment, and this led some FSWs to hide the bottle when taking it.

“Yes, I take it with me in the bag. I have a friend who said you are HIV positive after looking at the tablet, then I told her that it is a preventive medicine, but she couldn’t believe me. She is also a sex worker, so I brought her to the DIC and they told her everything. Then after checking for liver and HIV, she also started taking it. When she first said that I was positive, I was shocked and started to hide when I take it. The drug container is similar to ART, so at first, you might shock.” [P 03, age 23]

#### Feeling challenged by adherence needs

As with any other medicine, maintaining good adherence to the PrEP was mentioned as one of the challenges, especially since the FSW’s job involves substance use and going far from home. Also, the working hours mostly are at night, so most FSWs spend the morning time sleeping, which makes FSWs who take in the morning forget to take it.

“I drink alcohol and chew Khat, it is not advised to drink with the drug and that worries me. When it’s cold, I drink to cope. Sometimes I sleep and forget to take it; especially if you take it in the morning, you could forget.” [P 12, age 25]

The size of the pills was also mentioned as a challenge. One FSW said that the drug looks like a tablet which is given for cows in the countryside; you have to use more water to swallow it.

## Discussion

The study explored the reasons behind condom failure and the mechanisms FSWs use to minimize the harm, including acceptability of and usage of PrEP. The results show that despite FSWs’ struggles to ensure proper condom use, condom failure remains a challenge, both due to the clients’ behavior and factors related to their own inadequacies. When condom failure occurred, FSWs tried to minimize its adverse consequences with various strategies, and some, but not all, FSWs used PrEP. PrEP acceptability was compromised by different factors including fear of the side effects and doubts as to the effectiveness of protection. Those who had started taking PrEP reported various challenges including adverse side effects, stigma, and problems with maintaining adherence.

A common occurrence was that client’s behavior and demands created uncomfortable situations for FSWs, sometimes leading to deliberate condom breakage/slippage. Studies in Kenya and India also reported that clients who prioritized sexual pleasure purposely interfered with condom use [10, 18, 19]. Similar to the current study, a qualitative study conducted in Kenya also mentioned that condom breakage/slippage could either be intentional or unintentional [22]. Besides the deliberate act, due to the influence of alcohol or other substance use, clients could compromise condom use unintentionally. Therefore, becoming aware of client-related factors that lead to condom failure could help FSWs manage their own protection better.

Although FSWs attributed most of the breakages to the actions of clients [22], FSWs’ actions and negligence also caused condom failure. Similarly, findings from a study in India showed that FSWs under the influence of alcohol could not control condom use, and therefore, the failure rate could be high [23]. In addition, as found in the current study, their emotional involvement with the sexual intercourse could increase the friction leading to breakage. Also, when they became too actively involved or absorbed in the sex, their ability to manage and feel condom breakage/slippage could be compromised. Having sex in a position where FSWs cannot control the actions of the client also opens the door for condom failure. Therefore, programs need to make a special effort to identify FSWs who appear to be more susceptible to breakage/slippage and provide information and skills to minimize their risk for HIV/STI infection [13, 20]. Although they work under difficult conditions, FSWs try to maintain their safety by controlling the condom use process. In addition, as shown in the current study and in a review of studies conducted among FSWs, some took PrEP to be protected from HIV during condom breakage/slippage incidents [24].

PrEP is one of the prevention methods that was recently introduced in Ethiopia [14]. Following Ethiopian national comprehensive HIV prevention, care, and treatment guidelines, the Ministry of Health in collaboration with partners has started PrEP as an additional HIV prevention service for FSWs [14]. The continuous risk of condom failure shown in the current study suggests that PrEP may play a key role in HIV prevention strategies, if uptake among FSWs could be ensured. However, our study identified several misconceptions with regard to the prevention benefits of PrEP which could have implications for PrEP uptake. FSWs reported that they thought PrEP was an alternative to condom use, and some FSW preferred using condoms rather than PrEP. Some even compared taking PrEP daily with taking ART for HIV treatment. Such misconceptions might be due to poor comprehension of the part of the FSWs or poor communication skills from the provider side. Therefore, PrEP providers should be aware that may FSWs have a low level of education and will need engaging information sessions to fully appreciate the value of PrEP, especially as misconceptions regarding side effects persist in the community. To support PrEP uptake and persistence, the toolkits should be easily understandable by FSWs [25]. Studies conducted in Baltimore and South Africa also suggested the need for provision of proper PrEP education to increase the uptake [21, 23].

The disbelief in the effectiveness of PrEP was also another reason to avoid PrEP, and it is consistent with prior research conducted among young adults in rural Kenya, Uganda and Botswana [25, 26]. In the aforementioned study, some of the individuals wanted to see evidence if PrEP really works [26]. In addition, taking the pills daily was mentioned as a burden, indicating that a low level of information might cause a barrier for PrEP uptake. There is an urgent need to provide PrEP information among FSWs including how PrEP should be taken and adhered to. Strategies should be designed for how to communicate proper information about PrEP with the aim of changing the attitude of FSWs towards PrEP.

Our study also showed that preconceptions and lack of confidence played a role in avoiding PrEP. Some anticipated that their drinking and Khat chewing status might interfere with PrEP, and others mentioned that PrEP taking could make them negligent about condom use. Such cases illustrate that the role of service providers is vital. Service providers should anticipate such issues and discuss how to overcome fear and use PrEP for protection against HIV. On the other hand, FSWs who were taking PrEP mentioned that taking it improved their condom use since it boosted their confidence in protection. Therefore, creating a peer to peer education program to allow these two different attitudes to meet and discuss could help to strengthen PrEP programming for FSW.

On the other hand, our study showed that FSWs who were taking PrEP also faced various challenges which emanated from community perceptions about PrEP. A study conducted in Uganda reported that stigma related to the similarities of PrEP with ART made some FSWs fear taking PrEP, because they could be perceived as if they had HIV [26]. Due to that, some FSWs try to hide when taking it, which eventually affects their adherence. Systematic reviews of studies conducted in low and middle income countries reported that adherence could be impacted by the complexity of the FSW’s lifestyle [27]. They mostly work at night, might use substances, and thus spend the morning time sleeping. Those who start taking it therefore could forget taking it on time and could even forget taking it at all. Studies conducted in South Africa, Benin, Senegal, and Uganda reported a lower retention rate of PrEP, especially among young FSWs, which could be attributed to similar challenges like substance use [17, 28–30]. Although not conducted among FSWs, studies targeting couples across Africa found that women who had experienced verbal, physical or economic abuse from a partner were more likely to have low PrEP adherence [31–34]. It is known that most FSWs experience abuse, frequently exposing them to stress and forgetting [31, 34]. Therefore, PrEP education programs should target common challenges associated with PrEP use in order to support uptake and adherence.

Furthermore, the study identified several side effects that not only were considered negative by those who had started taking PrEP but also created fear among those who did not take it. Service providers should make FSW aware that side effects will be temporary and usually disappear within two weeks. In general, good and ongoing counseling is key for successful PrEP service roll-out [24, 35]. It helps to increase enrollment, maximize adherence and reduce attrition for the success of the service. Service provider’s knowledge and skills need to be built through ongoing mentoring and training based on evidence from studies such as the current one and others [23].

### Methodological considerations

The study was conducted using an in-depth interview which allowed participants to express their experiences and concerns. We used a private venue for the interviews to create an environment where the participants could freely discuss personal issues. The trustworthiness of the study was enhanced through mutual discussion of the analytical process with all authors, which helped to achieve consensus and to ensure the credibility of the results. In addition, the data collection was conducted by two experienced interviewers from Ethiopia.

The presentation of the various steps in the analytical model makes the process accessible to other researchers and readers of the paper. In addition, the use of quotations in the text provides evidence that the interpretation of the findings was based on the current data.

The study has some limitations. Although the data collection process was finalized after saturation of information was achieved, it cannot be excluded that additional participants might have provided other perspectives. The small number of interviews conducted and the fact that the study was done in only one location might limit the transferability of the findings. However, the study was conducted in Addis Ababa which is the capital city of Ethiopia with the largest number of FSWs when compared to other cities. Although the findings might not reflect the perspectives of all FSWs, the challenges faced by FSWs would generally be similar throughout the country. The fact that most FSWs working in different settings face similar challenges makes the results of the current study in all probability transferable to most FSWs in other settings and countries.

Most of the participants were from one type of sex selling venue (street) which might introduce some limitations. The views and attitudes of FSWs from other types of venues might be missed. However, to our knowledge, this is the first study that explores the uptake of PrEP among FSWs in Ethiopia. It may serve as the basis for future studies and may offer suggestions for strengthened PrEP education programs for FSW.

## Conclusion

Despite FSWs’ efforts to minimize the risk of HIV/STI through proper condom use, factors that increase their exposure to condom failure remain, including the demands and/or behavior of the clients and FSWs’ own actions and/or poor awareness. Challenges associated with one of the harm-reducing mechanisms, PrEP uptake, indicate that user-friendly strategies need to be designed and implemented to counter these barriers and facilitate PrEP uptake. There is a need for programs to consistently support FSWs’ struggle against HIV infections. Programs should alert FSWs that PrEP is a method that they can control, which can support the condom use process. The information provided to FSWs should take into consideration their education and understanding to achieve better HIV prevention strategies.

## Supplementary Information

**Additional file 1.** The In-depth interview guide used for data collection.

## Data Availability

All data generated or analyzed during this study are included in this article.

## Abbreviations

FSW: Female sex workers
STI: Sexually transmitted infection
HIV: Human immunodeficiency virus
HED: Heavy episodic drinking
PrEP: Pre-exposure prophylaxis
ART: Anti-retroviral treatment
PSI: Population service international
NGO: Non-governmental organization
DIC: Drop-in centers
