# Supplementary material for: Condom failure and pre-exposure prophylaxis use experience among female sex workers in Ethiopia: a qualitative study
Source: BMC Public Health. 2022 May 31;22:1079. doi: 10.1186/s12889-022-13468-3 (PMC9158269; doi:10.1186/s12889-022-13468-3)
Supplement: Supplementary file 1 — Additional file 1. The In-depth interview guide used for data collection. [file 12889_2022_13468_MOESM1_ESM.docx]

In-depth Interview Guide English

**Introduction:** Hello my name is ------- and as per the consent you have given I would like ask you few questions. Thank you in advance for your participation.

**Objective:** To explore the experience of FSWs with regard to risky sexual behavior, PrEP use and strategies to minimize the risk.

Therefore the interview guide will try to explore

1. What makes you decide to use or not use condom

- Being under the influence of Alcohol/drug( either the FSW or the client)
- Money issue
- Violence (the client) or other etc…

1. What strategies you use to overcome/minimize the risk (to minimize sex without condom, which will minimize the exposure to STI/HIV)
2. PrEP use experience

Interviewer Initials: ______________

Date: __________________________

Start time ______________________

End time: _______________________

**Participant information**

Study number: _________________

Age: ____

**Background**

- Can you tell me about yourself and where you come from.

**Probe:** Marital status? Has Children?

- Can you tell me a bit about your schooling history
- Please tell me how you started getting money in return for sex?

**Probe:** Age, marital status, prior work experience,

**General Introduction**

- Selling sex can be a risky business; can you tell me your thoughts about this?
- What sort of worries do you have, and how do you deal with them?
- Can you tell me about a typical risky situation? How do you deal with that sort of situation?
- What do you experience as the most difficult challenge with regard to protecting your own health?

**Clients**

Next, I would like to discuss about your clients and some challenges you may have experienced, including physical violence.

- Can you tell me a bit about your clients? Their life status, work etc..?
- What do you usually look for - in a client?

**Probe:** tell me Examples of client you have said ‘no’ to? And Examples of clients you wanted to say ‘no’, but didn’t? Describe.

- Tell me about any experiences you might have had of being forced to have sex? What was the reason?

**Probe:** Threatened, Beaten, Raped by a client?

- Have you ever heard about women being raped or beaten doing this work? What do you think was the reason
- What do you do to avoid clients that you do not want?

**Condom use**

In the next section, I’d like to discuss about condoms use and challenges:

- Can you tell me about using condom with your clients?

**Probe:** Advantage? disadvantages?

- How do you handle a situation related with condom?

**Probe:** slipping or tearing/breakage? How do you think it happened? How common is that? Did you continue using condom despite the slippage or tearing? any other problems?

- How do you decide whether to use a condom or not with a client’s?

**Probe:** what sort of situations might make you reluctant to use condoms?

- How do you negotiate about using condoms with a client; how do you make sure they are used properly?

**Probe:** Who normally suggests using a condom? Who buys? Who puts on? Who takes off? Anything else important to consider?

- How does the type of relationship you have with your customer affect the use or non-use of condom?
- What is your experience with a client who does not want to use condom?

**Probe:** Are there differences with different kinds of sex acts? If you want to use, how did you convince them? What happened when you refuse to have sex without condom?

- Tell me about the time when you wanted to use a condom but didn’t? What was the reason?

**Probe:** condom access**,** fear of the client**?**

**Alcohol**

Next, I would like to discuss about alcohol consumption and challenges

- Can you tell me about the influence of alcohol drinking during sex work

**Probe:** can you tell me your experience after having excessive alcohol? Can you tell me your experience with a drunken client? How does it affect their sexual act?

- What do you do to minimize excessive alcohol drinking?

**Probe:** if you drink too much, if the client drinks too much?

- Can you tell me how you manage safe sex after you drink or with a drunken client?
- What kind of support do you think that help you to minimize excessive alcohol use?

**Probe:** from whom?

**Health**

Next, I would like to discuss about health issues and some challenges you may have experienced.

- Can you tell me about the potential health risks connected with your work?
- Can you tell me about your STI infection experience?
- How do concerns about STI affect your work condition?

**Probe:** How do you minimize your risk? What resources are available? How accessible are they? How do you use them? Example?

- How much do you worry about HIV? How serious do you think HIV infection is these days?

**Probe:** How do sex workers you know minimize their risk? How do you minimize your risk? How concerns about HIV do affected your work? Describe.

**Question on PrEP**

- What do you know about PrEP?
  - Prevention vs treatment
  - How does it work?
- Would you please describe your experience being on PrEP?

**Probe:** stigma; fear of PrEP safety; misconceptions, other?

- Did you experience any side effects? How did you cope with them?

**Probe:** Call the clinic? Came back to the clinic? Consulted a friend?

- What worries do you have in taking PrEP?

**Probe:** concerns such as frequency risky (condom less) sex; fear for stigma/discrimination;

- Do you have problems that influence your adherence? What would you say could help you to better adhere to PrEP?

**Probe:** alcohol use, drug abuse; time spent at the clinic;

- What has been the most challenging for you taking PrEP?

**Closure**

In general is there anything that you want to discuss, that was not covered during our conversation? Or is there anything you would like to add?”

**Conclusion: Thank you very much for your time and for talking with me.**
